# MMP-9 Levels and IMT of Carotid Arteries are Elevated in Obese Children and Adolescents Compared to Non-Obese

**DOI:** 10.5935/abc.20170025

**Published:** 2017-03

**Authors:** Claudio Andrade, Adriana Bosco, Valeria Sandrim, Francisco Silva

**Affiliations:** Santa Casa de Misericórdia de Belo Horizonte - Núcleo de Pós-Graduação e Pesquisa, Belo Horizonte, MG - Brazil.

**Keywords:** Pediatric Obesity, Biomarkers, Atherosclerosis, Tissue Inhibitor of Metalloproteinase

## Abstract

**Background:**

Childhood obesity is associated with increased risk of atherosclerosis and cardiovascular disease in adulthood. Increased intima-media thickness (IMT) of the carotid artery is linked to the initiation and progression of the chronic inflammatory processes implicated in cardiovascular disease. Matrix metalloproteinase-9 (MMP-9) plays an important role in the degradation of the extracellular matrix and, consequently, in the development, morphogenesis, repair and remodeling of connective tissues.

**Objectives:**

(i) to determine and compare the concentrations of MMP-9, tissue inhibitor of metalloproteinase -1 (TIMP-1), and MMP-9/TIMP-1 ratio in obese and non-obese children and adolescents; (ii) to investigate the association of these markers with common and internal IMT of carotid arteries.

**Methods:**

Cross-sectional study involving 32 obese and 32 non-obese (control) individuals between 8 - 18 years of age.

**Results:**

Significantly (p < 0.05) higher values of MMP-9 concentration, as well as a higher MMP-9/TIMP-1 ratio were detected in the obese group compared to control counterparts. Common and internal carotid IMT values were significantly higher (p < 0.001) in the obese group compared to the control group. Positive correlations were observed between the common carotid IMT values and MMP-9 concentrations as well as MMP-9/TIMP-1 ratio.

**Conclusions:**

Our data demonstrate that obese children and adolescents present higher mean IMT values, plasma MMP-9 and MMP-9/TIMP-1 ratio compared to the non-obese. Thus, these findings indicate that this group presents a risk profile for early atherosclerosis.

## Introduction

Childhood obesity is a major health problem because of its association with an increased risk of atherosclerosis and cardiovascular disease in adulthood.^1^ Obesity is correlated to an increased intima-media thickness (IMT) of the carotid artery, which, in turn, is linked to the initiation and progression of chronic inflammatory processes implicated in cardiovascular disease.^1-7^ The increase in carotid IMT starts during childhood,^8,9^ and nearly all children present fat deposits in these arteries by the age of three.^10^ A study by Dawson et al.,^11^ with 635 adolescents and young adults, has shown that carotid IMT is significantly correlated to coronary artery risk scores; therefore, early assessment of this parameter through non-invasive methods may assist in the identification of individuals most at risk of cardiovascular disease.

Matrix metalloproteinase-9 (MMP-9) plays an important role in the degradation of the extracellular matrix and, consequently, in the development, morphogenesis, repair and remodeling of connective tissues.^13,13^ Since MMP-9 activity is regulated primarily by tissue inhibitor of metalloproteinase-1 (TIMP-1), an imbalance between MMP-9 and TIMP-1 could lead to the uncontrolled degradation of extracellular matrix as seen in various pathological disorders, including cardiovascular diseases.^13,14^ Thus, some studies in adults have correlated IMT values and circulating MMP-9/TIMP-1 concentrations;^15,16^ however, to our knowledge, no study has evaluated these correlations in children and adolescents. Also, increased IMT values of the carotid artery are linked to chronic inflammatory processes in cardiovascular disease,^1-7^ and this process involves the activation of MMP-9.

Therefore, we hypothesized that obese children and adolescents present higher concentrations of plasmatic MMP-9 and MMP-9/TIMP-1 ratio compared to the non-obese group, and that these concentrations are positively correlated to IMT values of common and internal carotid arteries. Thus, the aim of this study was to compare plasma MMP-9 and TIMP-1 levels and correlate these concentrations to IMT values of common and internal carotid arteries in obese and non-obese children and adolescents.

## Methods

### Study population and experimental design

Details of the cross-sectional study were presented to and approved by the Ethics Committee of the Hospital *Santa Casa de Misericórdia* in the city of Belo Horizonte (Belo Horizonte, MG, Brazil). Written informed consent was obtained from all participants and/or their legal guardians prior to the investigation.

Potential participants were recruited in the outpatient clinic of the Division of Endocrinology and Metabolism of *Santa Casa de Misericórdia* in the city of Belo Horizonte and included males and females between 8 and 18 years of age. Individuals presenting hypertension, metabolic, endocrine, autoimmune, neoplastic and infectious diseases were excluded from the study. Participants were assessed as obese (n = 32) or non-obese (n = 32; control group) according to their body mass index (BMI) referenced against the 2000 Centers for Disease Control and Prevention (CDC) sex-adjusted BMI-for-age growth charts with the cut-off point for obesity taken as ≥ 95^th^ percentile.^17,18^ Hypertension was defined by the "*VI Diretrizes de Hipertensão Arterial da Sociedade Brasileira de Cardiologia*" (VI Arterial Hypertension Guidelines from the Brazilian Society of Cardiology) and for children and adolescents, it was based on percentiles. Obese and non-obese groups were not on any medication. A minimum sample size of 23 individuals per group was calculated considering an alpha error of 0.05% and a test power of 90%. Data were collected between March 2010 and March 2012.

### Anthropometrical, clinical and biochemical evaluations

Anthropometrical (weight, height and BMI), clinical (carotid IMT) and biochemical (TSH, MMP-9, TIMP-1, MMP-9/TIMP-1 ratio) parameters were collected for all selected individuals. Anthropometric measurements were performed with participants barefoot and with light clothes. Body weight was measured using portable digital scales (capacity 180 kg; sensitivity 100 g), while height was determined by portable stadiometer (non-extendable 2 m measuring tape graduated in 0.1 cm divisions) with the subject in the orthostatic position. Systolic (SBP) and diastolic (DBP) blood pressures were measured at least three times after 15 min of rest and hypertension was defined as SBP and/or DBP exceeding the 95th percentile.^19^

Serum TSH was estimated with a commercial enzyme-linked immunosorbent assay (ELISA) kit (Quibasa Química Básica, Belo Horizonte, MG, Brazil). Plasma was collected in tubes containing EDTA as anticoagulant, MMP-9 and TIMP-1 tests were performed using human MMP-9/TIMP-1 complex DuoSet kit (R&D Systems, Minneapolis, MN, USA).

### IMT measurements

Common carotid artery: average measurement of the thickness on both sides, longitudinal projection, exactly 1 cm before the bifurcation. Internal carotid artery: average measurement of the thickness on both sides, longitudinal projection at the origin.

Measurements were performed using a Vivid i (GE Healthcare, Milwaukee, WI, USA) portable ultrasound system with the subject lying in the supine position and with the neck rotated (45º) to the side opposite to the undergoing examination.^20^ All examinations were performed by a single physician with certified skills in diagnostic imaging.

### Statistical analysis

Statistical analyses were performed with the aid of SPSS version 20.0 (SPSS Inc., Chicago, IL, USA). Student's t test was used to compare the mean values of the two groups regarding variables that were normally distributed, while the Mann Whitney test was used to compare variables that were not normally distributed. The χ^2^ test was employed to assess the relationship between carotid IMT and independent variables. The correlations among plasma biomarkers and common and internal carotid IMT were analyzed using Spearman's correlation. In all tests, statistical significance was set at 5% (0.05).

## Results

Clinical and biochemical characteristics of subjects enrolled in study are shown in Table 1. Although both groups exhibited serum TSH values within the normal range, the mean value of this parameter in the obese group was significantly higher (p < 0.05) than that recorded in the non-obese group (2.7 ± 0.8 *vs* 2.0 ± 0.8 µIU/mL, p < 0.05). Plasma MMP-9 concentrations were significantly higher in the obese group compared to the non-obese group (p < 0.05), while plasma TIMP-1 concentrations were similar (p > 0.05) in both groups. Mean MMP-9/TIMP-1 ratio was significantly higher (p < 0.05) in the obese group in comparison to the non-obese. Mean IMT values of the common and internal carotid arteries of obese individuals were significantly greater (p < 0.001) than those of their control counterparts.

**Table 1:** Demographic, anatomical and biochemical characteristics of obese and non-obese children and adolescents recruited in the outpatient clinic of the Division of Endocrinology and Metabolism of *Santa Casa de Misericórdia de Belo Horizonte* (Belo Horizonte, MG, Brazil).

Variable	Obese group [n = 32]				Non-obese group [n = 32]			
	Minimum	Maximum	Mean/%	SD	Minimum	Maximum	Mean/%	SD
Age [years]	8	17	13	2	12	18	15*	2
Height [m]	1.28	1.79	1.57	0.13	1.52	1.84	1.63*	0.08
Weight [kg]	47	120	73	17	35	71	56*	9
BMI [kg/m^2^]	26	40	29	5	15	23	22*	2
SBP (mmHg)	90	120	103	6	90	110	103	6
DBP (mmHg)	50	70	60	7	50	80	63	7
Gender (% Female)	-	-	59	-	-	-	47	-
TSH [µIU/mL]	1.5	4.6	2.7	0.8	0.7	4.2	2.0*	0.8
Common carotid IMT [mm]	0.38	0.58	0.45	0.04	0.38	0.45	0.42*	0.02
Internal carotid IMT [mm]	0.36	0.46	0.42	0.03	0.37	0.44	0.40*	0.02
MMP-9 [ng/mL]	127	1208	343	249	92	925	246*	151
TIMP-1 [ng/mL]	322	1165	677	214	207	1522	709	284
MMP-9/ TIMP-1 ratio	0.15	1.47	0.48	0.25	0.11	1.59	0.41*	0.31

BMI: body mass index; SBP: systolic blood pressure; DBP: diastolic blood pressure; TSH: thyroid-stimulating hormone; IMT: intima-media thickness; MMP-9: metalloproteinase-9; TIMP-1: tissue inhibitor of metalloproteinase-1; SD: standard deviation.

* Significant differences p < 0.05 compared to obese group.

**Figure 1:**
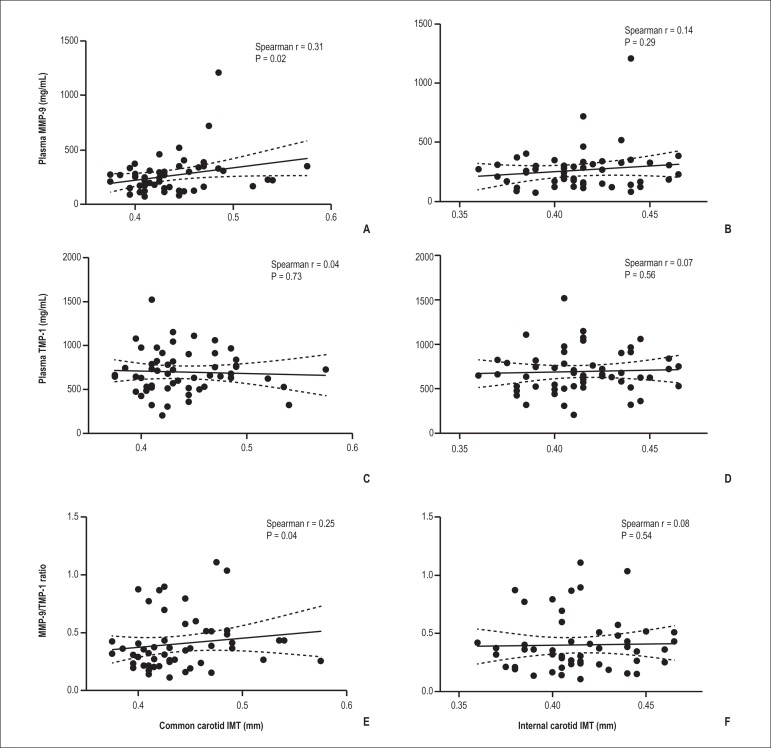
Correlations among biomarkers [MMP-9 (A,B), TIMP-1(C,D) and MMP-9/TIMP-1 ratio (E,F)] and common (A,C,E) and internal (B,D,F) carotid IMT. The correlations among plasma biomarkers and common and internal carotid IMT were analyzed using Spearman's correlation.

There was a direct and statistically significant correlation among plasma MMP-9, MMP-9/TIMP-1 ratio, and IMT values of the common carotid artery (p = 0.02 and p = 0.04, respectively: Figure 1, A and E). In contrast, there was no significant correlation between plasma TIMP-1 and IMTs of common and internal carotid arteries (Figure 1, C and D) or MMP-9 and IMT of internal carotid arteries (Figure 1B).

## Discussion

To our knowledge, this is the first study to correlate plasma MMP-9 and TIMP-1 levels to common and internal IMT in obese and non-obese children and adolescents. Following an evaluation of matrix metalloproteinases in obese and non-obese children and adolescents, Glowińska-Olszewska et al.^12^ reported high concentrations of the atherosclerosis marker MMP-9 in the obese group and even higher concentrations in hypertensive obese individuals. These authors argued that the abnormally high concentrations of MMP-9 could indicate modifications in the metabolism of the extracellular matrix of blood vessels and heart muscle, and that such alterations could speed up the atherosclerotic process. Additionally, the same research team described that MMP-9 and TIMP-1 concentrations were elevated in obese children and adolescents, and that the values of these parameters increased even further when obesity was accompanied by hypertension.^12^ Moreover, Belo et al.^21^ reported that genotypes and haplotypes of MMP-9 gene modulate circulating MMP-9 levels in obese children and adoslecentes. In the present study, plasma MMP-9 and the ratio MMP-9/TIMP-1 were significantly higher in obese individuals compared to their control counterparts, but the two groups presented no statistical difference in plasma TIMP-1. Although weak, it was possible to demonstrate a direct relationship between the concentrations of MMP-9 and MMP-9/TIMP-1 ratio, but not those of TIMP-1 and IMT values of common carotid arteries, suggesting a potential participation of this gelatinase in artery remodeling. Furthermore, no such relationship could be established with internal carotid IMT. This difference of correlations could be explained by the magnitude of the IMT of the internal carotid that is lower than that of the common carotid; therefore, the difference of magnitude may have interfered in the correlation. It is important to note that plasma MMP-9 concentrations reflect the systemic MMP-9 production and not only the vascular production, which may reduce the magnitude of correlations between this biomarker and IMT.

In the present study, mean IMT values of the common and internal carotid arteries of the obese group (0.47 and 0.43 mm, respectively) were significantly increased (p < 0.001) compared to those of the control group (0.42 and 0.40 mm, respectively); a result that is in agreement with previous reports.^22,23^ Thus, in a case-control study carried out in Belgium by Beauloye et al.,^23^ involving healthy subjects between 8 and 18 years of age, the mean value of carotid IMT of the obese group (0.470 mm) was significantly greater than that of the non-obese control group (0.438 mm), even though the mean age of the two groups did not differ significantly. Furthermore, these authors were able to demonstrate a significant positive correlation between carotid IMT and relative BMI. Moreover, studying Brazilian adolescents, Silva et al.^24^ demonstrated, in 35 obese and 18 non-obese subjects between 10-16 years old, that cIMT, triglycerides, HOMA-IR, insulin, and CRP values were higher, while high-density lipoprotein cholesterol (HDL-c), adiponectin, and VO_2max_ values were lower in the obese group than in the non-obese group.^24^

Based on mean IMT values of the common carotid artery determined in the obese and control groups in the present study, a cut-off point of 0.44 mm was established. A sonographic evaluation of common carotid and femoral arteries of 247 healthy subjects between 10 and 20 years of age^25^ revealed that mean IMT values increased almost linearly from 0.38 to 0.40 mm with increasing age. Since the adopted cut-off point was considerably higher than the value previously ascribed to healthy individuals in the age range 18 to 20 years of age, it is possible to state that children and adolescents comprising the obese group in the present study exhibited abnormally increased carotid IMT values. Moreover, it was possible to estimate from the data obtained that the risk of the obese group exhibiting elevated common carotid IMT was 2 to 5 times higher than that of the control group, while the risk of increased internal carotid IMT was 1.5 to 4 times greater.

Non-invasive techniques are reliable tools for identifying adults with increased risk of atherosclerosis and cardiovascular risk, but for children and adolescents, such techniques have been reserved mainly for research purposes. Ultrasound imaging appears to be a reliable technique to estimate IMT values of human arteries *in vivo*, since Pignoli et al.^26^ were able to confirm that there were no significant differences between B mode-determined IMTs of the common carotid arteries evaluated in pathogenic examination and those evaluated *in vivo* in young subjects. Moreover, while the analysis of IMT has often been used in cross-sectional studies, only a few clinical trials with children have employed this parameter.^20^ The Cardiovascular Risk in Young Finns study,^27^ which comprised a 21 year follow-up longitudinal investigation, suggested that obesity indices, such as BMI, skinfold, serum lipoproteins, insulin, glucose and blood pressure, measured in youth, are significantly associated to increased IMT and decreased elasticity of the carotid artery in adulthood. These findings emphasize the importance of weight control from youth to adulthood in reducing cardiovascular risk.

Although mean TSH value of the obese group was higher than that of the control group (2.85 versus 1.98 µIU/mL), no cases of hypothyroidism were diagnosed in obese participants. Conventionally, a serum TSH concentration of 4 to 5 µIU/mL is considered elevated; however, recent data from large population studies have indicated that a lower TSH cut-off point in the region of 2 to 2.5 µIU/mL would be more appropriate.^28^ Likewise, the National Academy of Clinical Biochemistry has recommended an upper limit of 2.5 µIU/mL^29^ for serum TSH, a value that is below the mean concentration of the obese group determined in the present study. However, it is not possible to state with certainty that cases of subclinical hypothyroidism were absent within the obese group of the present study.

In addition, numerous studies have revealed a positive association between measures of obesity and serum thyroid-stimulating hormone (TSH) concentrations, although the mechanisms responsible for this association require further elucidation,^30^ it is proposed that variations in thyroid hormone could affect lipoproteins and oxidation steps contributing to vascular remodeling and endothelial function.^31^ Interestingly, a significant correlation has also been demonstrated between carotid IMT and TSH values within normal reference values, suggesting an increased cardiovascular risk in subjects with low normal thyroid function.^31^

Yap and Jasul^32^ found a positive correlation between serum TSH and BMI, and inferred that an increase in TSH concentration, even within the generally accepted limits, could contribute to weight problems. The present study demonstrated that the group of obese children and adolescents exhibited increased TSH concentrations, although the concentrations were within the normal range, similarly to findings previously reported by Aypak et al.^33^ However, this problem clearly requires further investigation since hypothyroidism may be associated with markers of atherosclerosis and, consequently, with increased carotid IMT.^34,35^ A limitation of our study is the small number of subjects enrolled.

## Conclusion

Our data demonstrate that obese children and adolescents present higher mean IMT values, plasma TSH, plasma MMP-9 and MMP-9/TIMP-1 ratio compared to the non-obese. Thus, these findings indicate that this group presents a risk profile for early atherosclerosis.
